# Ionic Losses and Gains in Perovskite Solar Cells: Impact on Efficiency and Stability

**DOI:** 10.1021/acsenergylett.5c02435

**Published:** 2025-09-08

**Authors:** Miguel A. Torre Cachafeiro, Wolfgang Tress

**Affiliations:** † Institute of Computational Physics, 30944 Zurich University of Applied Sciences (ZHAW), 8400 Winterthur, Switzerland; ‡ Institut des Matériaux, École Polytechnique Fédérale de Lausanne (EPFL), 1015 Lausanne, Switzerland

Mobile ionic charges can significantly affect the performance of perovskite solar cells (PSCs) in terms of power conversion efficiency (PCE), as well as stability if their density changes over time. However, assigning performance losses during aging to ionic losses requires a reference point which is independent of the mobile ion density. In this Viewpoint we discuss, using drift-diffusion simulations, how the mobile ion density can affect the PCE obtained from a fast *J*–*V* scan when the preconditioning voltage does not match the voltage at which the ionic space charge vanishes. Depending on device properties, this misalignment can induce relative performance gains thanks to mobile ions and lead to an overestimation of the ionic loss. Taking into account the interplay between ion density and competing recombination pathways is crucial for the loss and degradation analysis of PSCs.

## Ionic Losses as the PCE Difference between the Fast and Slow *J–V* Scan

Over aging of PSCs, the PCE measured fast from an open-circuit voltage (*V*_OC_) precondition often shows far less degradation than the stabilized PCE.^1–3^ This has been attributed to the slow response and electric field screening effect of mobile ions,^4^ which aggravates as the ion density increases over time, affecting mainly the short-circuit current (*J*_SC_).^1,2,5–8^ Recent studies using scan rate-dependent *J*–*V* measurements and drift-diffusion simulations have shown that the PCE from a fast *J*–*V* scan starting with a *V*_OC_ precondition, can reflect the stability of a device where ionic screening is prevented.^2,7,9^ If the effective built-in voltage (*V*_bi_) in the perovskite layer is high enough that *V*_OC_ *≈ V*_bi_, fixing the ionic distribution for the *V*_OC_ value can approximate what would happen in an equivalent device without ions.^10,11^ This is because the ionic charge should be mostly spread throughout the bulk, where negative and positive ions would compensate each other. Thus, the PCE from a fast *J*–*V* scan where ions have no time to respond (starting from a precondition around *V*_OC_) can be used as a reference point from which to measure the ionic loss as

1 $$\mathrm{Ionic loss}≔\Delta \mathrm{PCE}={\mathrm{PCE}}_{\mathrm{fast}}-{\mathrm{PCE}}_{\mathrm{stabilized}}$$

Any losses also present in the preconditioned PCE_fast_ can then be attributed to non-ionic losses, e.g., increase in nonradiative recombination.^2,3,9^ Nonetheless, analyzing performance losses this way across all PSCs in general requires caution, as ions may not always be the culprit.^12,13^ If the electrostatic effect of the ion density *N*_ion_ alone can significantly decrease the stabilized PCE of a device, as observed during degradation,^2^ it means that diffusive electron and hole transport is not efficient and the device requires the assistance of the bulk electric field to extract the photogenerated charges.^14^ In short, a high sensitivity of the *J*_SC_ to *N*_ion_ alone requires pre-existing recombination pathways which compete with diffusion-dominated transport.^15^ However, initial ion densities in solution-processed perovskites are expected to be high,^16,17^ meaning that substantial field screening could in many cases be present from the start. Thus, it is arguable whether high-efficiency PSCs should be generally so sensitive to the electrostatic effect of *N*_ion_ alone, or if first the recombination rate should increase sufficiently for the available *N*_ion_ to amplify any losses. However, the interplay between ions and recombination, determining whether their presence amplifies or minimizes losses relative to an ion-free device, depends strongly on the energetic configuration of the PSC.^12,13^

## Energetic Alignment Dominates Ionic Impact on PCE

Figure 1a,b shows two energetic configurations for comparison (simulation parameters are provided in Table 1). In the first case, the high difference in the work function of the electrodes and aligned charge transport layers (CTLs) result in a high *V*_bi_ in the perovskite layer. As shown in Figure 1a, the “ion-free” voltage nearly coincides with the 1 sun-*V*_OC_ of the device (1.2 V), lying just above it (1.25 V). However, lowering the work function difference and introducing an energy offset with the CTLs shifts the “ion-free” voltage significantly below *V*_OC_ (0.85 V), as shown in Figure 1b. This difference critically affects the accuracy of disentangling ionic from non-ionic losses using the described method (eq 1). For context, “ion-free” voltages for high efficiency p-i-n PSCs were recently reported in ref 18, comparing various perovskite compositions and self-assembled monolayers (SAMs); resulting values range from 0.6 to 1.0 V, consistently below the *V*_OC_ of the devices (by different amounts).

**Figure 1:**
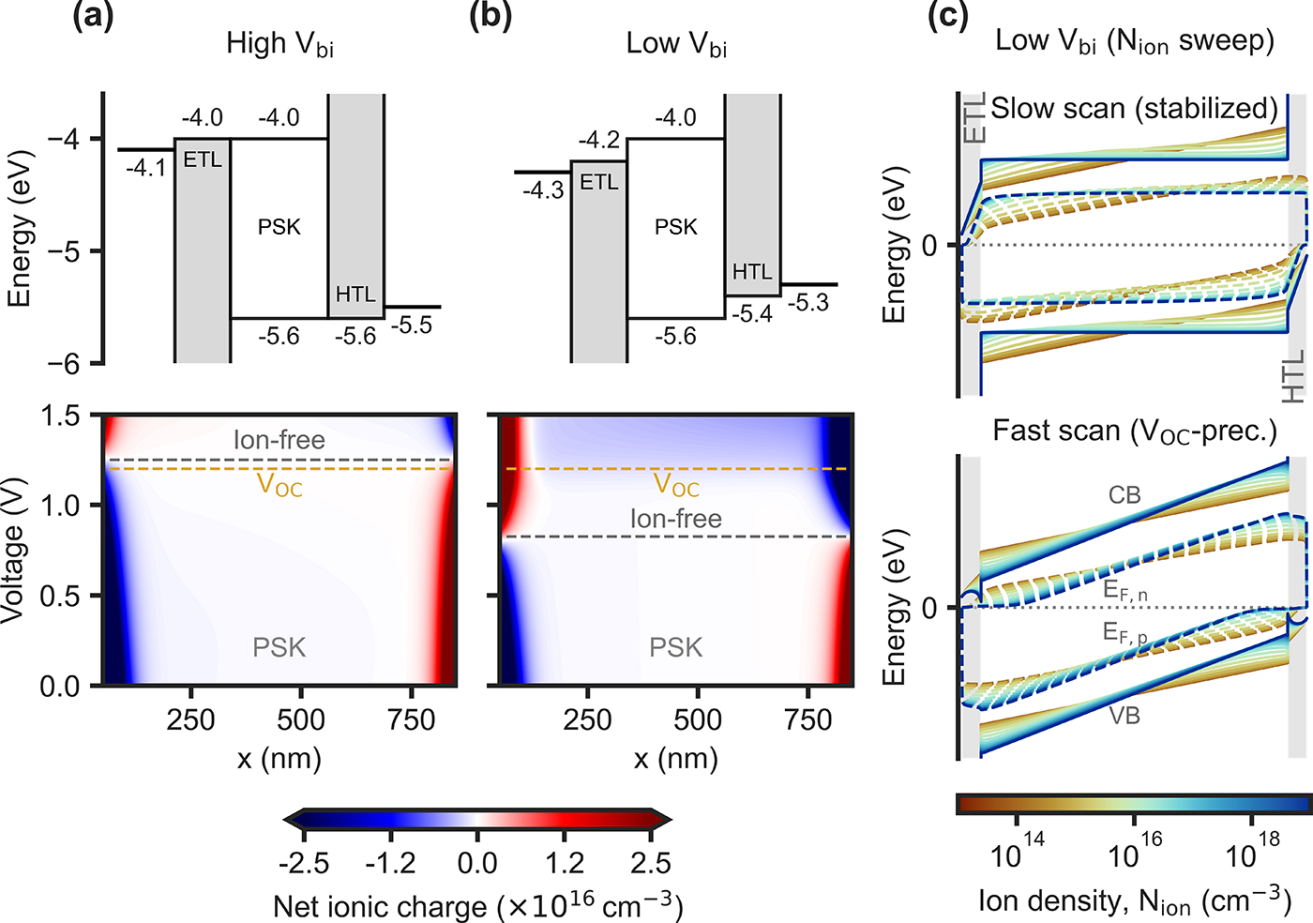
Energy band configurations for PSC with (a) aligned CTLs and high *V*_bi_ and (b) offset CTLs and lower *V*_bi_, showing the respective net ionic charge profiles under different applied voltages, for an input *N*_ion_ of 10^17^ cm^–3^. The dashed lines highlight the 1 sun-*V*_OC_ and the voltage where the net ionic charge has its minimum (“ion-free”). (c) Simulated band diagrams at short circuit for the low *V*_bi_ case with varying *N*_ion_, showing the stabilized condition and the profiles where the ionic charge is instead fixed at the *V*_OC_ value in (b).

**Table 1:** Parameters Used in the Simplified PSC Model in Setfos^a^

Parameter	**ETL**	**PSK**	**HTL**
Thickness, *d* [nm]	50	800	50
Valence band edge, VB [eV]	-7.5	-5.6	-5.4
Conduction band edge, CB [eV]	-4.2	-4.0	-2.1
Density of states, DOS_VB_ [cm^*–*3^]	10^19^	10^19^	10^19^
Density of states, DOS_CB_ [cm^*–*3^]	10^19^	10^19^	10^19^
Electron mobility, μ_n_ [cm^2^ V^*–*1^ s^*–*1^]	10^–3^	1/Varied (**Sweep 1**)	10^–3^
Hole mobility, μ_p_ [cm^2^ V^*–*1^ s^*–*1^]	10^–3^	1/Varied (**Sweep 1**)	10^–3^
Anion mobility, μ_a_ [cm^2^ V^*–*1^ s^*–*1^]		Uniform & static	
Cation mobility, μ_c_ [cm^2^ V^*–*1^ s^*–*1^]		5 × 10^–10^	
Dielectric constant, *ε*	5	25	5
Anion density, *N*_a_ [cm^*–*3^]		**Sweep**	
Cation density, *N*_c_ [cm^*–*3^]		**Sweep**	
Bimolecular rec. prefactor, β_γ_ [cm^3^ s^*–*1^]		10^–10^	
SRH lifetimes, τ_n_ and τ_p_ [ns]		200 (Sweep 1)/*∞* (Sweep 2)	
Interface rec. velocity, *v*_int,SRH_ [ms^*–*1^]		0.1 (Sweep 1)/Varied (**Sweep 2**)	

a The electrode work functions are given in Figure 1. ETL and HTL denote the electron and hole transport layers, respectively. The energetic alignment corresponds to the lower *V*_bi_ case. The energy level values for the high *V*_bi_ case are given in Figure 1a. The generation rate in perovskite is calculated using AM1.5G spectra, the complex refractive index of MAPbI_3_ and common CTL materials from the Setfos database.

Figure 1c shows simulated band diagrams at short circuit for the low *V*_bi_ model, with varying the ion density *N*_ion_. The lowest *N*_ion_ considered is sufficiently low to be effectively the same as an equivalent device without ions.

At short circuit, the reduced band tilt in the perovskite shows that the electric field decreases due to the ionic space charge accumulated at the contacts. For the high *V*_bi_ case, fixing the ionic charge at the *V*_OC_ distribution prevents the screening effect, restoring the “ion-free” band diagram. However, for the low *V*_bi_ case, ions are not any more compensated in the bulk at *V*_OC_, and the ionic space charge layers invert polarity relative to the short circuit distribution (Figure 1b); as a result, increasing *N*_ion_ now tends to strengthen the bulk electric field, as seen by the steeper tilt of the bands. This introduces a key implication: the fast *J*–*V* scan from a *V*_OC_-precondition is no longer independent of *N*_ion_, so it cannot be used as reference to classify losses as ionic or non-ionic.

## Ideal Case (High *V*_bi_): Ionic Losses = ΔPCE

To discuss the interplay between ionic and electronic effects, we look at a rising *N*_ion_ with surface recombination as an example, which is a known limiting loss in PSCs.^19^ Figure 2 shows simulated PCE values for varying the interface Shockley-Read-Hall (SRH) recombination velocity (*v*_int,SRH_) with varying levels of *N*_ion_, for the high *V*_bi_ case (Figure 1a) where the initial *V*_OC_ (1.2 V) roughly matches the “ion-free” voltage. The simulation parameters are specified in Table 1. The difference between the stabilized PCE (Figure 2a) and the preconditioned PCE where the ionic distribution is always fixed for the initial *V*_OC_ value (Figure 2b), is shown in Figure 2c. An example degradation pathway where both *N*_ion_ and *v*_int,SRH_ increase is shown by the gray dashed lines, and plotted in Figure 2d. As expected, in this case the analysis based on eq 1 works nicely, since the preconditioned PCE mostly follows the ion-free trace, which directly corresponds to the non-ionic loss. For this example, the ionic loss (eq 1) would be only slightly underestimated, due to the slight misalignment between the precondition and the “ion-free” value in Figure 1b. Ideally, the preconditioned PCE in Figure 2b would be constant vertically, for the fast scan to be fully independent of *N*_ion_. Then, if ΔPCE is not constant horizontally (Figure 2c), it means the ionic loss is being amplified by a deteriorated electronic property. This is further illustrated by a sweep of the CTL mobility (Figure S1 in the Supporting Information), demonstrating how a limited charge mobility in the CTLs^20,21^ can also result in an increased ionic loss. Furthermore, if Figure 2b is not mostly constant vertically, then the ionic loss does not correspond to ΔPCE.

**Figure 2:**
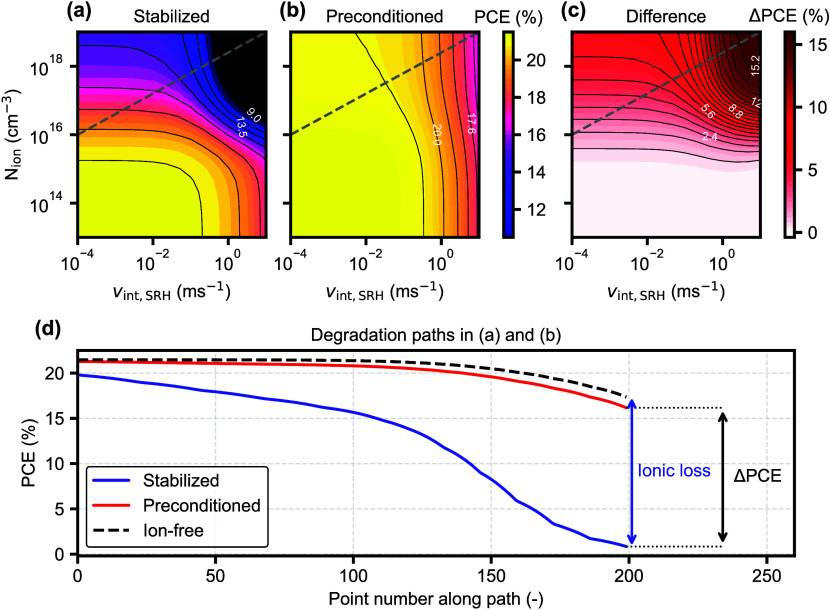
Simulation for PSC with aligned CTLs and high *V*_bi_. (a)–(c) Simulated PCE maps as a function of *N*_ion_ and interface recombination velocity *v*_int,SRH_, from stabilized and preconditioned *J*–*V* curves. (d) Example degradation pathways as shown by the dashed lines in (a) and (b), where both *N*_ion_ and *v*_int,SRH_ increase.

## Low *V*_bi_ Case: Ionic Loss ≠ ΔPCE

Depending on the energy level alignment, *V*_OC_ can be higher than the effective *V*_bi_,^11,13^ leading to ionic space charge accumulation also at *V*_OC_ (Figure 1b). With effective *V*_bi_ we mean the potential drop in the perovskite, which can be lower than the work function difference of the contacts due to high potential drop in the CTLs, especially when they are undoped and have a low dielectric constant. If there is ionic accumulation at the precondition used for the fast scan, the resulting *J*_SC_ may then benefit from the increased electric field in the bulk provided by the ionic space charge, which increases with *N*_ion_ as shown in Figure 1c. Such accumulation can also have a negative effect depending on the device properties and available recombination pathways, potentially leading to inverted hysteresis,^22,23^ which could result in a negative ΔPCE.

To exemplify how ionic accumulation may affect the analysis, we look at the low *V*_bi_ case, where the “ion-free” voltage is lower than *V*_OC_. Two scenarios are compared; in the first one, denoted as “Sweep 1”, the electronic mobility in perovskite μ_PSK_ is varied for different *N*_ion_ (mimicking degradation in the charge transport properties of the perovskite), with unmodified bulk- and interface-SRH recombination parameters. In the second scenario (“Sweep 2”) there is no bulk-SRH, and only the interface SRH-recombination velocity *v*_int,SRH_ is varied for different *N*_ion_ (as in the first example above), keeping a constant μ_PSK_. The simulated performance metrics of the PSC under varying *N*_ion_ are shown in Figure S2 in the Supporting Information. For Sweep 1, reducing μ_PSK_ provides a higher sensitivity to changes in the electric field. At any value of μ_PSK_, the charge collection efficiency drops due to the screening effect of *N*_ion_, reducing the *J*_SC_. However, the *V*_OC_ and fill factor (FF) show an inverse dependence and actually benefit from the ionic space charge, as explained in refs. 12,13 As a result, while the PCE tends to reduce with decreasing μ_PSK_, it first increases with *N*_ion_ until μ_PSK_ is sufficiently low. For Sweep 2, a similar trend can be observed with *v*_int,SRH_. Here, the initial PCE (low *v*_int,SRH_) is also initially higher with enhanced *N*_ion_, again due to the increase of *V*_OC_ ^24^ and FF dominating over the loss in *J*_SC_. The trend in PCE with *N*_ion_ then inverts once *v*_int,SRH_ is sufficiently high.

Thus, in simulations the stabilized PCE of a PSC is not always higher in the absence of mobile ions, since for instance, they can enhance the cell’s tolerance to energetic offsets with the transport layers.^12,13^ Figure 3 compares how the PCE differs from the stabilized one when preconditioning above the “ion-free” voltage and fixing the ions in the resulting distribution. The preconditioned PCE again corresponds to the fast scan PCE measured in experiment, where ions have no time to respond. The effect of ionic accumulation at the precondition voltage (1.2 V) is not independent of *N*_ion_, as the PCE is not constant vertically in Figure 3b. For both the μ_PSK_ sweep (Figure 3a–c) and the *v*_int,SRH_ sweep (Figure 3d–f) the preconditioned PCE is always higher than the stabilized one, as in the first example above. This results in a positive ΔPCE (eq 1) which increases with *N*_ion_, especially for high recombination rates, either due to the low μ_PSK_ or to the high *v*_int,SRH_. However, ΔPCE tends to saturate at moderately high *N*_ion_ once the electric field is completely screened, as seen by the mostly vertical lines in Figure 3c where *N*_ion_ ≳10^17^ cm^–3^. Most importantly, the preconditioned PCE becomes significantly more tolerant to higher recombination rates by increasing *N*_ion_, as seen by the horizontal shift of the PCE contours in Figure 3b,e in the high *N*_ion_ range. The ΔPCE maps in Figure 3c,f show that, along a horizontal path at constant *N*_ion_, ΔPCE increases significantly due to a change in electronic properties that ions amplify. This is similar to the analysis of *J*–*V* hysteresis, where a change in hysteresis does not necessarily indicate a change in ionic properties.^25^ Therefore, in practice it is important to complement ΔPCE analysis with techniques that can identify changes in *N*_ion_.^2,26^

**Figure 3:**
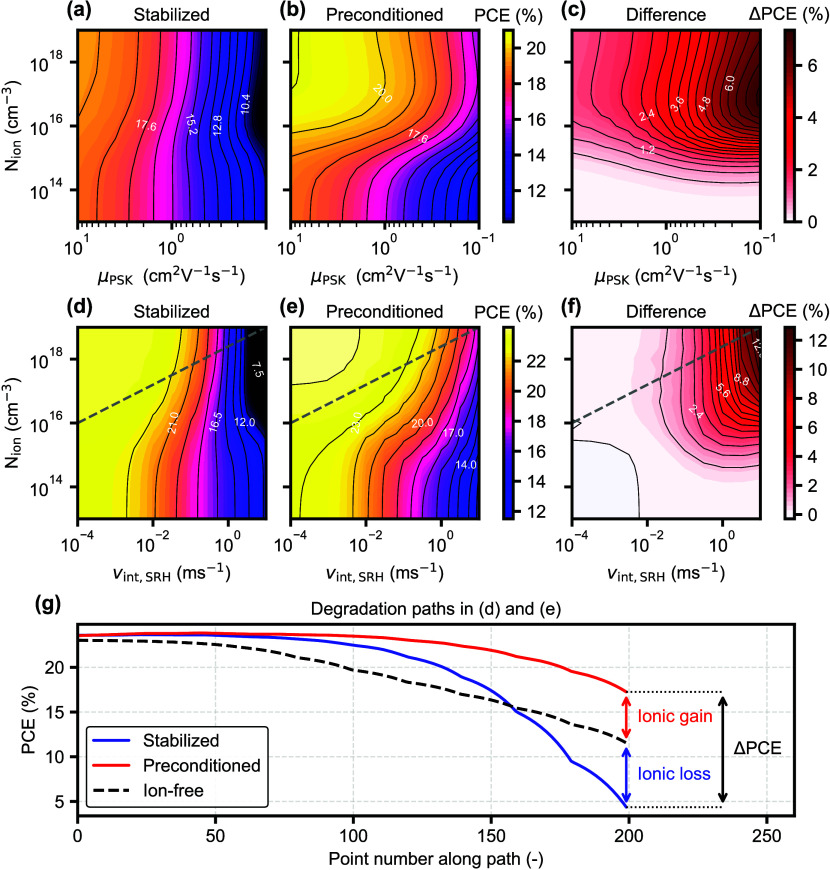
Simulation for PSC with energy offset at CTLs and lower *V*_bi_. (a)–(c) Simulated PCE maps as a function of *N*_ion_ and electronic mobility in perovskite μ_PSK_. (a) Stabilized PCE (slow *J*–*V* scan). (b) PCE with a fixed ionic distribution preconditioned at 1.2 V (fast *J*–*V* scan from a precondition around the initial *V*_OC_). (c) Difference between the preconditioned and stabilized PCEs. (d)–(f) Simulated PCE maps as a function of *N*_ion_ and interface recombination velocity *v*_int,SRH_, from stabilized and preconditioned *J*–*V* curves. (g) Example degradation pathways as shown by the dashed lines in (d) and (e), where both *N*_ion_ and *v*_int,SRH_ increase.

For the *v*_int,SRH_ sweep in Figure 3d–f, an example degradation pathway is drawn as a gray diagonal line across the *v*_int,SRH_–*N*_ion_ space. This would roughly correspond to a situation where both the ion density in perovskite and the quality of the interfaces are degrading over time. The PCE difference between the stabilized and preconditioned performance is shown in Figure 3g, which highlights how there can be an ionic gain relative to the ion-free performance. The trace of the ion-free device with increasing *v*_int,SRH_ can be assigned to the non-ionic loss, i.e., the degradation resulting from *v*_int,SRH_ alone in an equivalent device without ions. In the initial stages, there is a higher PCE for both the stabilized and the preconditioned curves, which means that there would be no real ionic loss at this point, despite the PCE difference that would be measured between a fast and a slow *J*–*V* scan. An ionic loss can be seen in the final stages, where the stabilized curve decreases below the ion-free trace. However, the ΔPCE between the preconditioned and stabilized traces still comprises both a gain and a loss relative to the ion-free performance, so the ionic loss from eq 1 would be overestimated. As a result, ionic losses can only be quantified correctly if the “ion-free” trace is known, as otherwise the PCE for a precondition around *V*_OC_ is also dependent on *N*_ion_. A PCE decrease where *V*_OC_ is mostly unchanged and a decreasing *J*_SC_ dominates is not either directly indicative of ionic changes alone, since in this case an increasing *N*_ion_ could compensate the radiative yield loss at *V*_OC_ that would be expected from a rising *v*_int,SRH_. In simulations, this can always be easily checked, and the equivalent device performance without ions should be reported when discussing an ionic loss. In experiment, fast *J*–*V* scans from the true “ion-free” precondition voltage (if it exists in practice and can be determined^11,13,18^ ) should be used as the reference PCE trace over aging.

## “Ion-Free” Voltage May Change during Degradation

As discussed above, ionic accumulation does not depend only on the potential originating from the work function difference between the electrodes, but also on the energetic offsets Δ*E*_CTL_ with the CTLs. So far we have considered an “ion-free” voltage which remains mostly unchanged over time, although it is possible that the degradation of CTLs, which often preludes that of the perovskite, could significantly shift this voltage over time. This could happen for instance, if the SAM in a p-i-n device degrades^27^ and loses its dipole. Figure 4a depicts the simulated net ionic charge for different energetic offsets Δ*E*_CTL_, where the dashed lines highlight the voltage at which most of the ionic charge is equilibrated in the bulk. Thus, using a constant preconditioning voltage for the fast scan PCE may result in a deviation from the “ion-free” condition, even if the starting one aligned. This would require a dynamic adaptation of the “ion-free” preconditioning voltage during aging.

**Figure 4:**
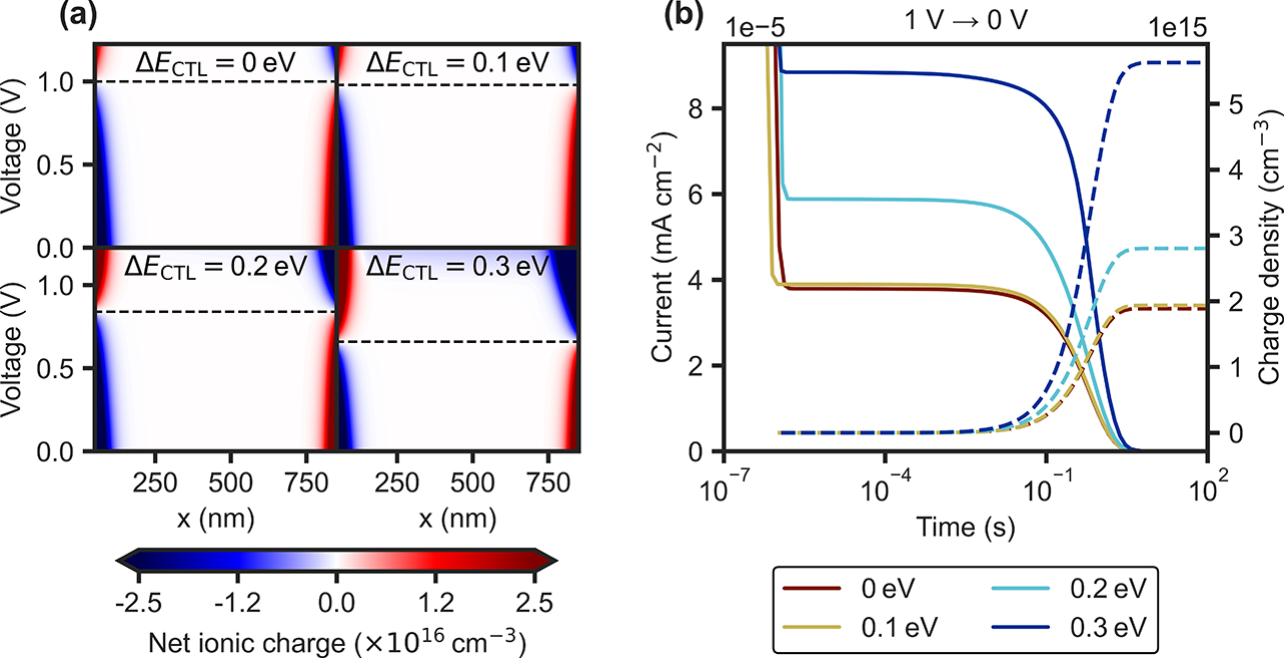
(a) Net ionic charge across the depth of the perovskite bulk (*x*) for different applied voltages and energy level offsets with the CTLs (Δ*E*_CTL_), for an input density of 10^17^ cm^–3^. The work function difference between the metal electrodes is kept constant at 1 eV. The “ion-free” voltage is marked as a dashed line for each case. (b) Transient dark current upon a sudden switch from 1 to 0 V, for the different Δ*E*_CTL_ configurations and cumulative ionic charge density using $N_{\mathrm{ion}}=\int J\left(t\right)\;dt\frac{1}{{q\,\;d}_{\mathrm{PSK}}}$ .

## Implications to Quantify Ion Density

Changed electronic properties can alter the level of ionic accumulation, for constant ionic properties (Figure 4a). This has implications for ΔPCE, but also for other measurements, as discussed next. To unravel how much of the performance changes originate from ionic electric field screening, it is important to quantify changes in *N*_ion_. A useful approach is measuring the dark transient current upon a change from a preconditioning voltage (e.g., 1 to 0 V).^28^ The drift of mobile ions after the sudden switch results in an external current which can be assigned to the ion density needed to screen the electric field,^29^ although slow electrochemical processes (e.g., defect self-healing^30^) may also contribute to the measured current at longer time scales.^31^ A rise in the transient current is expected to reflect a rise in *N*_ion_. In experiment, the dark current can increase moderately^7,8^ or even by orders of magnitude after stressing,^2,31,32^ although it saturates earlier in simulations.^26,33^ Without looking at the details of the sensitivity of this technique, which have been investigated in refs 26, 29, and 33, we look briefly at the effect of a changing “ion-free” voltage on the transient current response. If the transient current is measured always from the same precondition, 1 V in this example, and the “ion-free” voltage is shifting during degradation, this will already affect the amount of ionic accumulation at the precondition (Figure 4a). Thus, a change of the energetic alignment directly leads to a significant change in the current response (Figure 4b), even if *N*_ion_ remains constant (10^17^ cm^–3^ in this example). Therefore, while changes in the transient current may reflect changes in the amount of ionic accumulation, the underlying cause may not always be clear as any changes in the internal field resulting from CTL degradation can also have a significant effect on the ion migration current.^33^

## Disentangling Ionic and Non-ionic Losses

In simulations, high mobile ion densities which can screen the bulk electric field can in theory be mostly harmless for all device architectures, and even beneficial for some, as long as diffusion-dominated transport remains efficient. In this regard, it remains to be answered whether ionic and recombination properties can in practice remain so independent as done in simulations, or whether they are linked to a degree that preventing degradation of electronic properties requires mostly unchanged ionic properties also.^34^ The analysis of PCE degradation in PSCs should account for the interplay between different parameters, as illustrated here by the electrostatic influence of different ion densities on varying levels of competing recombination sources. In practice, electrochemical processes will also play a key role,^35^ determining the transient generation of ionic defects (and potential self-annihilation enabling reversibility),^30^ the mobility of those defects in different charge states,^36^ as well as their direct coupling to charge trapping and recombination. While it remains important to explore further experimental observations which require explicit ionic-electronic interactions in drift-diffusion models,^37^ simulations that consider only the electrostatic interaction of ions have so far reproduced many complex experimental trends successfully, and remain an important tool to understand the device physics of mixed ionic-electronic PSCs.

To summarize, the PCE difference (ΔPCE) between a fast and a slow (stabilized) *J*–*V* scan does not directly correspond to the ionic loss if there is ionic accumulation at the preconditioning voltage used for the fast scan. This point is important to consider regardless of whether the device benefits or suffers from the presence of mobile ions. While mobile ions can surely have a detrimental effect on PSCs, care should be taken not to blame them directly without sufficient evidence that the equivalent device without ions would perform better. In addition, non-ion-related parameters may amplify ionic losses; changes in electronic properties can increase ΔPCE, although all ionic parameters remain unchanged. Therefore, combining degradation analysis with measurements to probe any changes in ion density remains important. The fast PCE from a *V*_OC_ precondition, which can sometimes be characteristic of an “ionic gain”, should not be thought of as the performance of the equivalent device without ions, if the voltage at which ions are mostly compensated in the bulk is not known. Additionally, this value may also shift during degradation, which would require a dynamic adaptation of the preconditioning voltage used for the fast *J*–*V* scan, in order to properly disentangle ionic and non-ionic losses. Experimentally, it remains important to explore further characterization methods which can be used to determine the “ion-free” or “field-free” voltage and use them for degradation analysis. Taking this into account should help to establish a clearer picture of how mobile ions affect the efficiency and stability of PSCs.

## Supplementary Material



## Notes

Additional simulations (PDF)
