# Supplementary material for: Ionic Losses and Gains in Perovskite Solar Cells: Impact on Efficiency and Stability
Source: ACS Energy Lett. 2025 Sep 8;10(10):4849–55. doi: 10.1021/acsenergylett.5c02435 (PMC12519477; doi:10.1021/acsenergylett.5c02435)
Supplement: Supplementary file 1 [file nz5c02435_si_001.pdf]

Supporting Information (SI): Ionic Losses and Gains in Perovskite Solar Cells: Impact on Efficiency and Stability

Miguel A. Torre Cachafeiro[1,2] and Wolfgang Tress*[1]

[1]Institute of Computational Physics, Zurich University of Applied Sciences (ZHAW), 8400 Winterthur, Switzerland
[2]Institut des Matériaux, École Polytechnique Fédérale de Lausanne (EPFL), 1015 Lausanne, Switzerland

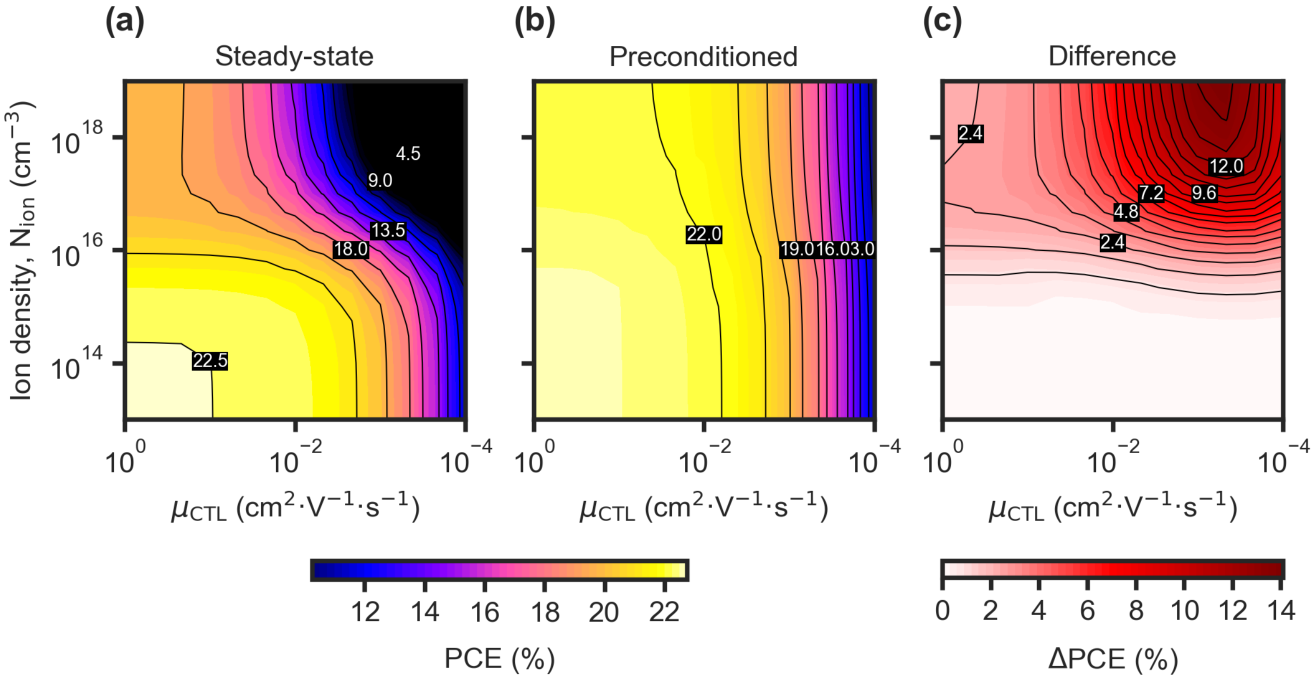

Figure S 1: Simulation for PSC with aligned CTLs and high V_{bi}. Charge transport layer-limited device, where the recombination rates are kept constant and only the mobility of electrons and holes in the CTLs (μ_{CTL}) is varied with N_{ion}. In this model the 'ion-free' voltage, where the absolute net ionic charge reaches a minimum, roughly coincides with V_{OC}. (a) Stabilized PCE and (b) preconditioned (1.2 V) PCE. (c) PCE difference between the preconditioned and stabilized values.

*Email: `wolfgang.tress@zhaw.ch`

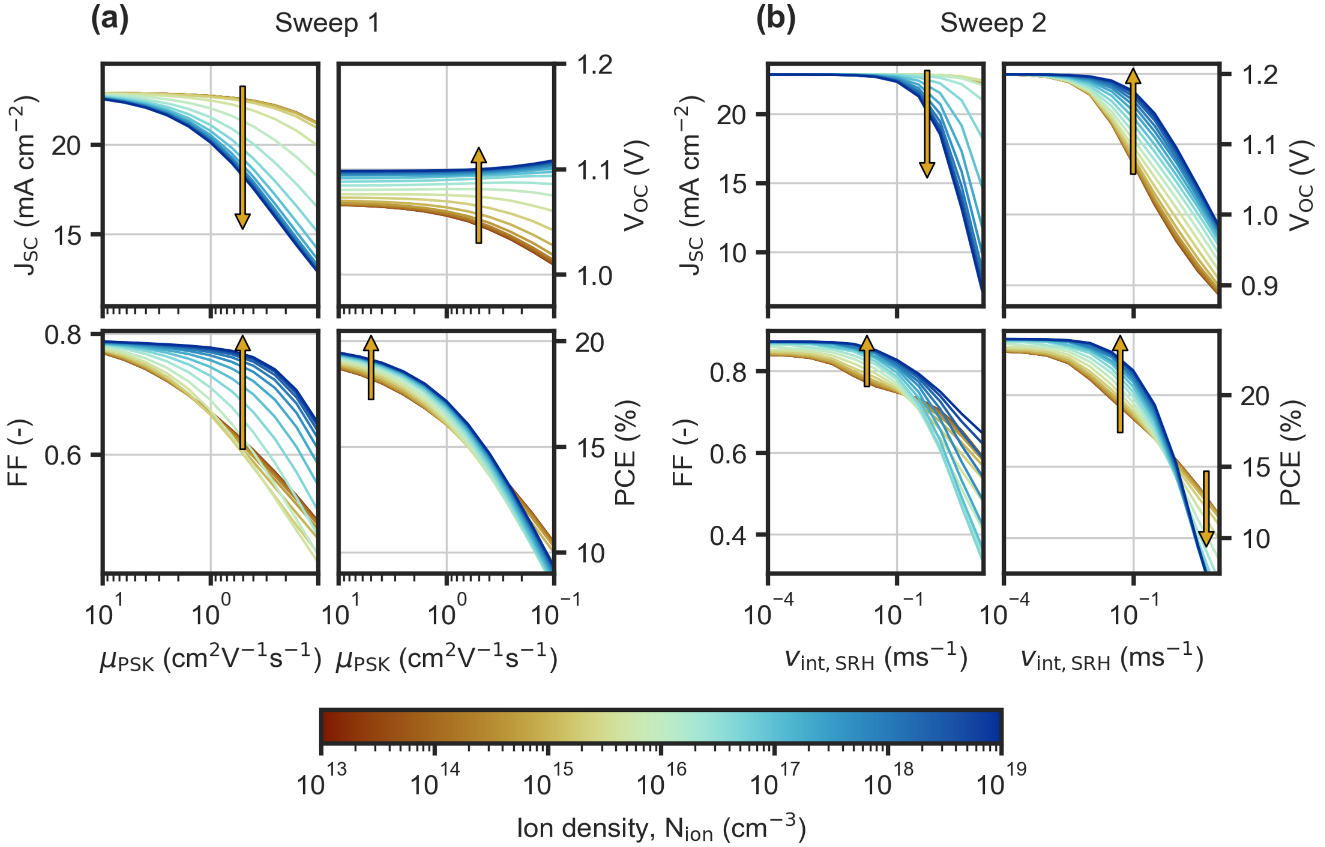

Figure S 2: Simulation for PSC with energy offset at CTLs and lower V_{bi}. Simulated stabilized J-V performance metrics with varying N_{ion}, where the arrows show the trend with increasing N_{ion}. In this model the 'ion-free' voltage, where the absolute net ionic charge reaches a minimum, is 0.85 V. (a) Bulk transport-limited device, where the recombination rates are kept constant and only the mobility of electrons and holes in perovskite (μ_{PSK}) is varied. (b) Device limited by the interfaces, where only $v_{int,SRH}$ is varied with N_{ion}.
